# Learning from end-of-life injectable medication patient safety incidents in the community: a mixed-methods analysis

**DOI:** 10.3399/BJGP.2025.0106

**Published:** 2026-01-13

**Authors:** Ben Bowers, Sioned Gwyn, Sarah Yardley, Stuart Hellard, P John Clarkson, Isobel J McFadzean, Kristian Pollock, Stephen Barclay, Andrew Carson-Stevens

**Affiliations:** 1 Primary Care Unit, Department of Public Health and Primary Care, University of Cambridge, Cambridge, UK; 2 Division of Population Medicine, Cardiff University, Cardiff, UK; 3 School of Health Sciences, University of Nottingham, Nottingham, UK; 4 Department of Engineering, University of Cambridge, Cambridge, UK; 5 Marie Curie Research Centre, Division of Population Medicine, Cardiff University, Cardiff, UK; 6 Queen’s Institute of Community Nursing, London, UK; 7 Swansea University, Swansea, UK; 8 Marie Curie Palliative Care Research Department, University College London, London, UK

**Keywords:** drug safety, end-of-life care, injections, patient safety, primary health care, subcutaneous

## Abstract

**Background:**

Processes to implement injectable end-of-life symptom control medications in the community are complex and can have an adverse impact on patient safety. Recurring patient safety incident types and their contributory factors remain underrecognised, inhibiting system-wide learning.

**Aim:**

To understand injectable end-of-life symptom control medication incidents, their contributory factors, the impact on patients/families, and identify priority areas for improving safe, effective, and timely care.

**Design and setting:**

Mixed-methods analysis of nationally reported injectable medication patient safety incidents involving adults in the community in England and Wales between 2017 and 2022.

**Method:**

A stratified random sample of 2150 incidents from the National Reporting and Learning System were screened for eligibility. Incidents that involved end-of-life injectable medications in the community were included and analysed. Deductive coding was undertaken to classify incident types, the contributory factors involved, the impact on the patient, and harm severity. An iterative thematic analysis was then conducted to identify patterns between recurring incident types and contributory factors.

**Results:**

In total, 419 patient safety reports detailed injectable medication-related patient safety incidents: 59.7% (*n* = 250) of incidents described harm to patients. Frequently reported patient safety incidents included: medication administration issues (49.2%, *n* = 206); delayed and inadequate assessments (10.3%, *n* = 43); and prescription issues (8.6%, *n* = 36). Incidents often involved multiple services and delays. Recurrent, and often interacting, contributory factors included inadequate continuity of care, distractions and mistakes, poor equipment design, and insufficient staffing levels.

**Conclusion:**

Interventions to improve injectable end-of-life symptom control care should focus on ensuring timely access to assessments and prescriptions, enhancing continuity of care, and mechanisms to ensure rapid visits to administer medication.

## How this fits in

The prescription and use of injectable end-of-life symptom control medications is a complex, risk-prone healthcare activity involving multiple services and actors. Yet, little is known about patient safety events involving these medications in the community. The present analysis highlights that reported incidents frequently involved delayed or inadequate assessment, prescribing, or medication administration issues. Key contributory factors included inadequate continuity of care, poor equipment design, insufficient staffing levels, and cognitive issues. Targeted system-wide interventions to improve end-of-life symptom management should focus on addressing these safety critical areas.

## Introduction

Effective, safe, and timely end-of-life symptom control in the community is a priority and source of concern for dying patients, their family carers, and healthcare teams.^1–6^ In 2022, 49.2% of all deaths occurred at home or in care homes in England and Wales.^7^ GPs have a leading role in prescribing injectable medications in advance of need or in response to worsening symptoms, including pain and breathlessness, nausea and vomiting, agitation, and respiratory secretions in the last weeks and days of life.^8–14^ These medications include opioids and sedatives, and are stored in the patient’s home with a ‘permission to administer’ chart, enabling a degree of clinical discretion in which drugs and doses are administered. Injectable medications are commonly administered by visiting nurses or paramedics when oral medications are impractical or ineffective, both as background symptom control and ‘as required’ injections.^15,16^ This is recommended and widespread practice in several countries, including the UK.^13,17–21^

The use of injectable medications at home is a risk-prone activity,^3,9,19,22–24^ involving several interacting components: decisions to prescribe; prescribing; dispensing; decisions to administer; administration; monitoring and adjusting treatment; post-death procedures; and medication disposal.^25,26^ These processes are multifaceted and complex, and are prone to adverse patient safety events, particularly as multiple people and services are involved.^9,12,15,19,27,28^ Recurrent safety issues, including difficulties accessing timely assessments and medication-related incidents, have been attributed to human factors, including mistakes and insufficient clinician skills, and structural conditions such as ineffective communication tools and inadequate staffing.^3,5,18,19,22–24,29^ It is distressing for all when care is perceived to be unsafe or patients are harmed by failures to achieve adequate symptom control.^5,22,23,29^

Patient safety incident reports provide valuable insights into how injectable medication-associated patient harms and near misses occur, as perceived and reported by clinicians.^30–32^ The National Reporting and Learning System (NRLS) was launched in England and Wales in 2004 to collate and enable learning from patient safety incidents. This is one of the largest incident databases worldwide, with all community-based NHS organisations providing data until it was superseded in 2023 by the Learn from Patient Safety Events service. NRLS data remain notably underutilised as a research resource to learn from end-of-life injectable medication patient safety incidents occurring in general practice and the community.^33,34^

The aims of this study were to analyse NRLS incidents occurring in the community to:

- understand injectable end-of-life symptom control medication incidents, their contributory factors, and impact on patients/families; and
- identify priority areas for improving effective, safe, and timely care.

## Method

### Study design

A mixed-methods analysis of nationally reported injectable medication patient safety incidents using the NRLS database for England and Wales was conducted.

### Data sources

Data were sourced from patient safety incidents reported between April 2017 and April 2022 that met the definition of *‘an event or circumstance that could have resulted, or did result, in unnecessary harm to a patient* [during healthcare delivery]*’*.^30^ These can be reported by any healthcare staff providing NHS care, regardless of setting, when they deem an incident has occurred. Reports included incident location, brief patient and incident characteristics, perceived harm severity, and unstructured free-text clinician narratives of what happened and why, plus plans to minimise the risks of reoccurence.^33^

### Study population, setting, and sample

The population were adults (aged ≥18 years) receiving end-of-life care in the community in England and Wales. Settings included general practices, out-of-hours services, ambulance services, pharmacies, learning disability services, palliative care, and nursing services. The free-text narrative of primary safety incidents for this population were searched using key search terms for palliative care and injectable end-of-life symptom control medications (see Supplementary Table S1), building on a previous search strategy.^34^ The search identified 45 590 potentially eligible incidents.

A stratified random sample of 2150 incidents was screened for eligibility. The sample size and composition were chosen to maximise understanding across a range of harm severities, within the capacity of the study resources. All incidents reported as resulting in death (*n* = 324) and severe harm (*n* = 334) were included (anticipating these may provide more detailed incident free-text narratives), along with a random stratified sample of moderate harm (*n* = 497), low harm (*n* = 497), and no harm (*n* = 498) incidents.

**Figure 1.**
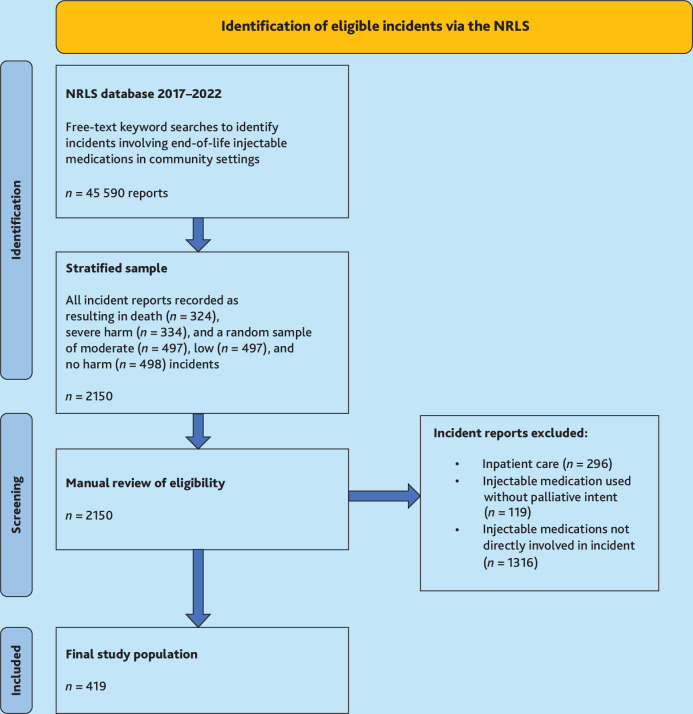
Flowchart of sample identification and screening. NRLS = National Reporting and Learning System.

Reports not describing patient safety incidents involving injectable end-of-life medications and those occurring in inpatient settings were excluded on manual review (Box 1). The first 20% of incidents were double screened by the first author (clinical academic community nurse) and the second author (clinical academic GP), with 94% concordance. Consensus decisions informed subsequent screening by the first author (Figure 1).

Box 1. **Inclusion and exclusion criteria**

**Inclusion criteria**

- Incident directly involved injectable end-of-life symptom control medications
- Patient aged ≥18 years
- Patient in last phase of life: report indicates that receiving (or should receive) palliative and end-of-life care
- Incident occurred in community settings, including transitions between care settings

**Exclusion criteria**

- Injectable medication used without palliative intent
- Injectable medications not directly involved in incident
- Patient aged <18 years
- Incident occurred solely in an inpatient care setting: hospital, hospice, mental health, and learning disability inpatient units
- Incident not related to patient care: did not describe a patient-level safety incident or near miss

### Data analysis

This mixed-methods analysis used the technique described as ‘following a thread’;^35^ quantitative findings informed qualitative lines of enquiry.

### Quantitative analysis

**Figure 2.**
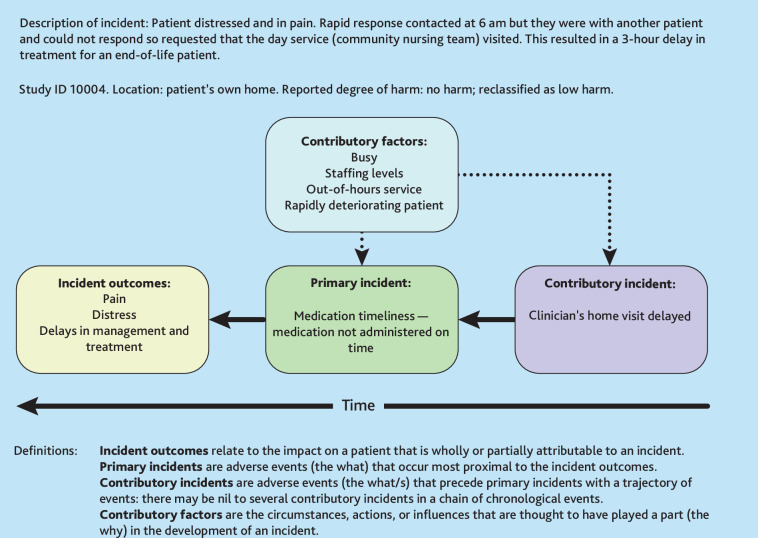
Recursive model of incident analysis example and definitions.^33^

Incident reports were first analysed using the Patient Safety (PISA) classification system, a coding framework aligned to theoretical concepts for learning from incidents within patient safety science.^30,33^ Codes within the framework are used in a deductive and recursive model of incident analysis.^36^ The PISA classification system was applied to categorise incident outcomes, primary incident types (most proximal to incident outcomes), contributory factors, and any preceding incidents (that is, the chain of events leading up to the primary incident) (Figure 2).

Harm severities were reclassified using the patient safety incidents in primary care criteria for consistency (see Supplementary Table S2).^37^

The first 20% of eligible incidents were double coded by the first and second authors, with consensus decisions informing subsequent coding by the first author. Descriptive analysis was undertaken to assess the most frequently harmful outcomes, primary incident types, and contributory factors. These findings informed the iterative qualitative analysis.^35^

### Qualitative analysis

An interpretive thematic analysis^38^ was used to identify recurrent patterns across primary incidents and contributory factors. After data familiarisation and reviewing the initial descriptive analysis, nuanced patterns and differences across incident types and narratives were identified by the first author. Memos were generated on recurring contexts and human and structural issues, along with how harms were presented and any evident key missing information.^38–40^ The reflexive, iterative decision was taken to group primary incidents by the most frequent clinical and medication management processes involved.^25^ All grouped incidents with free-text narratives containing ≥2 of the top 10 contributory factors were inductively coded, using NVivo (version 14), by the first author to identify and summarise relationships and interactions in incident contexts and the contributing and mitigating factors. Thematic patterns and variances across primary incidents and decisions in attributing significance to findings were discussed and refined with the second, fifth, seventh, eighth, and last author.

### Synthesis

Quantitative and qualitative findings were integrated to provide complimentary insights and identify priority areas for interventions.^41^ A driver diagram technique^42^ was used to map priority areas for improving safe and timely injectable medication care, informed by existing evidence for targeted interventions.

### Public involvement

The research was supported throughout by the study’s public and clinician advisory group: four family carers and two community nurses. Group members met together three times to discuss the study, help refine the driver diagram, and provide valuable, nuanced insights into the implications for practice.^43^

## Results

In total, 419 patient safety incidents were included. Incidents mainly occurred in patients’ own homes (74.7%, *n* = 313) or residential care homes (11.5%, *n* = 48) (Table 1). Opioids (44.4%, *n* = 186) and sedatives (such as, midazolam) (26.3%, *n* = 110) were the most commonly involved medications.

**Table 1.** Patient and clinical characteristics of the 419 incidents

Characteristic	*n*	%
**Age range of patients involved, years**		
18–65	91	21.7
66–75	89	21.2
76–85	117	27.9
≥86	122	29.1
**Place of incident occurrence**		
Home	313	74.7
Residential care home	48	11.5
Nursing home	20	4.8
Transfer between care settings	16	3.8
General practice surgery	8	1.9
Ambulance-based care	3	0.7
Community pharmacy	3	0.7
Out-of-hours service base	3	0.7
Other	5	1.2
**Medication involved^a^**		
Opioid	186	44.4
Sedative/anxiolytic	110	26.3
Antiemetic	59	14.1
Antimuscarinic	51	12.2
Non-opioid analgesia	2	0.5
Other	14	3.3
**Syringe driver involved**		
Yes	202	48.2
No	224	53.5
Unclear	3	0.7
**Harm severity (reclassified**)		
No harm/near miss	143	34.1
Low harm	143	34.1
Moderate harm	76	18.1
Severe harm	23	5.5
Death	8	1.9
Insufficient details to classify	26	6.2

^a^≥2 medications were involved in 94 incidents; 126 incidents did not indicate what drugs, if any, were involved.

Multiple medications were involved in 22.4% (*n* = 94) of incidents, although not all narratives indicated the drugs involved. Injectable medications were typically prescribed for use subcutaneously and 48.2% (*n* = 202) of incidents involved syringe drivers (continuous subcutaneous infusions delivered via a portable machine over 24 ho) (Table 1).

The study found 59.7% (*n* = 250) of reports described patient harm, 34.1% (*n* = 143) detailed no harm or near misses, and 6.2% (*n* = 26) provided insufficient detail to classify severity. Overall, 68 (16.2%) reports were labelled by reporters as ‘no harm’ incidents, yet physical and psychological harms to patients and their families were evident and incidents were reclassified for the study accordingly. Only eight (1.9%) incidents resulting in death were included: most were excluded as they were not about incidents relating to injectable medications or simply reported expected deaths (Table 1).

**Figure 3.**
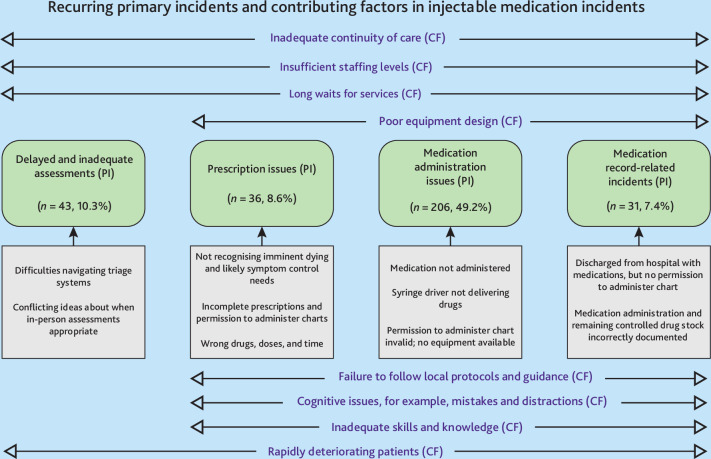
Process map of the recurring primary incidents and contributory factors in injectable medication incidents. CF = contributory factors. PI = recurring primary incident types.

Four main types of recurring primary patient safety incidents were identified: medication administration issues (49.2%, *n* = 206); delayed and inadequate assessments (10.3%, *n* = 43); prescription issues (8.6%, *n* = 36); and medication record-related incidents (7.4%, *n* = 31) (Figure 3). The most identified contributory factors across all incidents were: inadequate continuity of care (58.9%, *n* = 247); cognitive issues (39.9%, *n* = 167); rapidly deteriorating patients (30.8%, *n* = 129); and failure to follow local protocols and guidance (15.0%, *n* = 63) (Figure 3).

### Medication administration issues

Of primary incidents, 49.2% (*n* = 206) were medication administration issues. Patient outcomes included missed medication doses (*n* = 128), delays in treatment (*n* = 127), pain and discomfort (*n* = 90), agitation (*n* = 53), and disturbed dying — where patients were recorded as dying in considerable distress (*n* = 30).

Reports about medication not being commenced or administered in a timely way (*n* = 135) often detailed rapidly changing situations where medication could not be administered by visiting nurses as valid ‘permission to administer’ charts, equipment, or medication supplies were not available. Ineffective communication between healthcare teams was a frequent precursor to these primary incidents. Continuity of care and processes to check valid prescription charts and medication (re)supplies, where available, were compromised when multiple teams were involved and no single clinician or team led care. Families faced difficulties getting community nursing and hospice-at-home services to visit at weekends and resorted to calling emergency services:

Community nurse report: *‘Family have been trying to get hold of a healthcare professional for palliative care and support. Family state that they were not able to access anyone for the past three days. They have watched their relative deteriorate and be in pain. They have rung 999* [emergency services] *to give stat doses and* [hospice team] *refused to attend … Stat* [injection] *dose records were on* [ambulance trust] *follow up sheets for patients … Patient was clearly in last stages of their life.’* (Study ID 41002. Location: patient’s home. Reported degree of harm: no harm; reclassified as moderate harm)

Incident report narratives indicated unreliable processes for triaging and arranging urgent community nurse symptom control input visits; urgent messages were not given to the nurses allocated to visits. Further delays in symptom control care were mitigated when families or clinicians chased services:

Out-of-hours community nurse report: *‘Call placed at 5.00 am as palliative patient was in pain and agitated. Care staff and* [family] *were unhappy as they reported that they placed a call at 2.30 am and when no one had visited by 4.30 am they made contact again and the call had not been logged. When I visited patient at 7.00 am* [as I] *was held up with a previous visit, they informed me how long* [they] *had been waiting. Apologies given to both the* [family] *and care staff. Incident highlighted to call takers manager by email … Admin error.’* (Study ID 23012. Location: residential care home. Reported degree of harm: no harm; reclassified as low harm)

Issues in physically administering injectable medications were detailed in 71 reports. These included the wrong dose (*n* = 43) or wrong medication (*n* = 7) being given, with charts being misread because of distractions. Reports highlighted that clinicians were often working in stressful situations, with limited information about imminently dying patients. Not following local protocols and poorly designed ‘permission to administer’ charts contributed to incidents:

Agency community nurse report: *‘Call out to an end-of-life patient for a stat dose of pain relief by injection. I mistakenly took it from the dose stated for syringe driver which was* [fentanyl] *50 mcg. The dose should have been 25 mcgs, I gave twice the amount* [as an injection]*.’*

Manager adds to incident narrative*: ‘Important that staff are extremely careful when administering controlled drugs ... This is the second occasion recently when the administrating of medication has been taken from the wrong part of the palliative drug chart.’* (Study ID 17705. Location: residential care home. Reported degree of harm: no harm; reclassified as insufficient details)

Difficulties in delivering drugs via syringe drivers were commonplace (*n* = 58) and typically resulted in under- or overdoses of medication. Recurring issues included syringe drivers being incorrectly renewed, or the machine turning itself off in-between visits. This equipment required technical skill to set up, appeared poorly designed, and prone to settings being accidentally changed or malfunctioning silently:

Community nurse report: *‘Syringe driver replenished with patient’s medications, all okay, pump delivering, patient settled. Visit again today to replenish syringe driver and it was turned off and had only been delivering medications yesterday for around 1 hour so patient did not have any of the medication in the driver. Asked staff if they heard the alarm and they stated no ... Battery was replaced yesterday on visit and it was 100% and today it was down to 26%.’* (Study ID 31890. Location: residential care home. Reported degree of harm: no harm; reclassified as low harm)

Contributory factors in medication administration incidents included poor equipment design, mistakes and distractions, services being busy and insufficient staffing, inadequate skills and knowledge, reliance on agency staff, and inadequate continuity of care.

### Delayed and inadequate assessments

In total, 10.3% (*n* = 43) of incident reports described delayed and inadequate clinical assessments, often resulting in patients not receiving timely prescriptions of injectable medications. Patient outcomes included delays in management and treatment (*n* = 30), treating without sufficient information (*n* = 17), agitation (*n* = 9), and pain and discomfort (*n* = 8).

Insufficient referrals and communication between care providers, especially during out-of-hours periods and on hospital discharge, and difficulties navigating GP triage systems frequently contributed to delays and inadequate assessments. Patient harms were mitigated when nurses advocated for clinical reviews or sourced alternative prescriber reviews. It was typical for these incidents to be reported by community nurses; narratives often presented nurses’ frustrations about services not being accessible or responsive:

In-hours community nurse report: *‘Handover from night team to report that they had received a phone call from paramedics attending* [patient]*. Out-of-hours community nurse reported that she had requested a out-of-hours GP visit for* [patient] *for assessment and symptom management. Followed up on this request with a phone call to out-of-hours GP.* [GP’s initials] *did not at this point in time believe a home visit was required by [them]. I disagreed with this … Further discussion with* [another] *out-of-hours GP in the central dispatch team who re triaged and allocated for home visit … The just in case* [anticipatory] *medications supplied previously were out of date so no available medications to give.’* (Study ID 54400. Location: patient’s home. Reported degree of harm: severe harm; left as severe harm)

Conflicting ideas about appropriateness of assessments occurred between families and clinical teams. One family reported that an out-of-hours nurse was reluctant to visit and lacked compassion while the patient was agitated. This led to family members voicing concerns about symptom management and assessments in the future:

In-hours community nurse report: *‘*[Spouse] *and family were extremely distressed with the fact they had called out-of-hours as* [patient] *was extremely agitated. The* [family] *was called back 30 minutes later by who* [they] *think was a nurse, who stated that* [patient’s address] *is very far way, that* [they] *was all the way in* [another town] *and had* [they] *tried oramorph? …* [Family] *explained that the oramorph had not worked … In the end, the nurse stated* [they] *would visit, but the* [family should] *give the patient some oramorph, and if it calmed* [them] *down, to ring the out-of-hours back to cancel the visit. In the end, the* [family] *said forget it as* [they] *felt the nurse did not want to visit, and* [they] *did not want somebody* [they] *felt was not very compassionate to visit. Family extremely unhappy with how they were treated.’* (Study ID 80342. Location: patient’s home. Reported degree of harm: no harm; reclassified as low harm)

Contributory factors included inadequate continuity of care, rapidly deteriorating patients, insufficient staffing, services being busy with long waits for care, and disagreements among teams regarding priority and need for home visits.

### Prescription issues

Of primary incidents, 8.6% (*n* = 36) related to prescription issues. Patient outcomes included delays in management or treatment (*n* = 13), treating without sufficient information (*n* = 12), staff recognising mistakes and mitigating harms (*n* = 10), and pain and discomfort for patients (*n* = 6).

Anticipatory prescriptions were not completed appropriately in 24 incidents, often because of poor hospital discharge planning processes, or clinicians and teams not recognising likely symptom control requirements within the community:

Out-of-hours community nurse report: *‘Email received from Single Point of Access at start of night shift, sent at 3 pm* [regarding] *a patient who urgently needed controlled drugs and symptom control at home ... There was no* [permission to administer] *chart and anticipatory medications in place for symptom relief … The primary care team that includes the GP, palliative care, and community nursing services should have made better efforts to coordinate plans and put in place anticipatory medications, and not left to out-of-hours team.’* (Study ID 36491. Location: patient’s home. Reported degree of harm: low harm; left as low harm)

Other reports (*n* = 12) described prescription-related primary incidents. These included clinicians’ prescribing inappropriate medication intervals or dangerously high doses of opioids and sedatives. Conversely, when patients had previously been prescribed oral opioids, mistakes and omissions sometimes resulted in inappropriately low doses of injectable medications being prescribed. These were frequently identified and mitigated by clinicians before harm occurred. Patients experienced uncontrolled symptoms when prescription-related incidents were not prevented in time:

In-hours community nurse report: *‘Attended patient’s home for a crisis call. Patient is end of life and syringe driver was commenced evening of* [date: 2 days previously]*. Whilst assessing patient for rescue dose of morphine sulphate I discovered that the patient had been taking 60 mgs morphine sulphate twice daily orally. But that the syringe driver was written up to commence at 5 mg* [by out-of-hours GP]*.’* (Study ID 20343. Location: patient’s home. Reported degree of harm: low harm; reclassified as insufficient details)

Recurring contributory factors included inadequate continuity of care, cognitive mistakes, inadequate skills and knowledge, failure to follow local protocols and guidance, rapidly deteriorating patients, inadequate staffing levels, and stretched services.

### Medication record-related incidents

Medication record-related incidents were the primary incidents in 7.4% (*n* = 31) of reports, often affecting continuity of care. Patient outcomes included treating without sufficient information (*n* = 15), staff recognising mistakes and mitigating harm (*n* = 14), and delays in management and treatment (*n* = 7).

Eight primary incidents reported situations where patients were discharged home from hospital with injectable medications prescribed and issued, but no permission to administer charts had been completed to enable their administration. Community nursing teams mitigated risks by contacting GPs and out-of-hours doctors to complete charts. Mistakes in documenting medications administered and remaining stock occurred in 10 incidents, especially when staff or services did not use cross-organisational shared records. These incidents were identified at subsequent community nurse visits and required internal investigations to identify if controlled medication had gone missing:

Community nurse report: *‘I visited a patient to administer end of life medication, upon looking at the paperwork there was 1 less vial of each morphine and midazolam … Controlled drug sheet not completed, nil vials missing … Training issue identified … New preceptee* [nurse] *made aware of correct paper documentation.’* (Study ID 00286. Location: patient’s home. Reported degree of harm: no harm; left as no harm)

Contributory factors often included failure to follow local protocols and guidance, mistakes and distractions, inadequate continuity of care, agency staff, and new staff.

### Driver diagram

**Figure 4.**
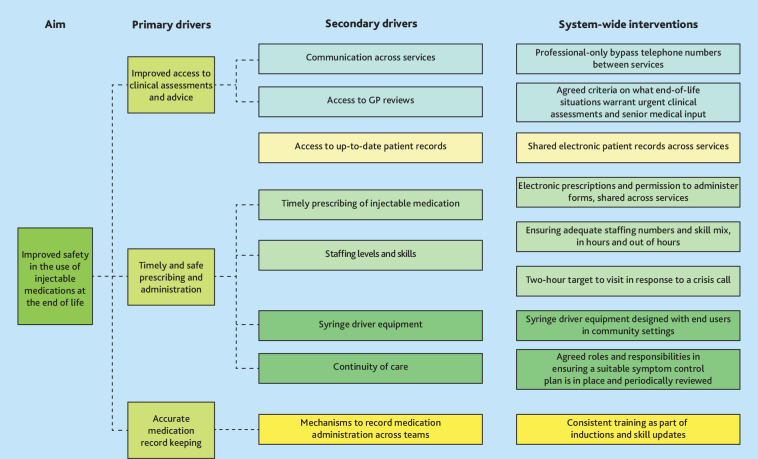
Driver diagram for system-wide improvement domains.

The study findings were used to identify priority areas for improving care: these were mapped into a driver diagram (Figure 4). This diagram highlights the data-driven primary and secondary drivers (improvement areas) requiring change, and evidence-informed system-wide interventions that could improve these elements (detailed on the right).

## Discussion

### Summary

Injectable medication-related incidents in the community often involved opioids, sedatives, and syringe driver equipment. Clinical assessment and prescribing issues, medication administration issues, and medication record-related incidents were frequently involved. Incidents occurred during in-hours and out-of-hour periods, often involving multiple services. The recurring critical and interacting contributory factors were inadequate continuity of care, cognitive issues, poor equipment design, insufficient staffing levels and long waits for services, failures to follow local protocols and guidance, and patients who are rapidly deteriorating.

The present findings highlight that system-level interventions offer considerable potential for improving end-of-life symptom control care: ensuring support for timely access to assessments and prescriptions; enhancing sharing of information; implementing mechanisms to support timely clinician visits to administer medication; and using user-friendly and reliable syringe drivers.

### Strengths and limitations

The stratified sampling approach enabled the authors to investigate a cross-section of incidents, reflecting a range of reported patient harms. This mixed-methods analysis using national data provided nuanced insights into patterns and recorded contributory factors and mitigating actions that influence unsafe care, including across different healthcare organisations and teams. Recurring issues were identified that can be hard to recognise and resolve at individual patient and service level.^28,39,44,45^

Patient safety incidents are underreported and any national incident database reliant on self-reporting risks an unrepresentative sample of patient harms and near misses.^31,33^ Report narratives reflect socially constructed healthcare practices in what gets detailed and omitted, and are dependent on clinicians’ interpretation of events at the time. Certain incidents are more likely to be reported, including those resulting in serious patient harms and consequences including death. Consequently, the authors have not made inferences about absolute numbers of injectable medication incidents occurring in the community and their causes. The present analysis helps in identifying priority areas for improving care and possible targeted interventions. The findings are currently informing longitudinal qualitative research investigating patients’, family caregivers’, and professionals’ experiences of safe and effective injectable medication care.

### Comparison with existing literature

Insufficient access to timely medical assessments have been repeatedly shown in qualitative studies to be a barrier to end-of-life prescribing.^46–49^ This is a key reason why the anticipatory prescribing of ‘as required’ injectable medications is advocated in research, international guidance, and expert consensus.^11,12,18,48,50–53^ Anticipatory prescriptions are frequently completed several weeks to months in advance of need, and require periodic review to ensure they remain clinically appropriate.^3,8,20,54^ The present study highlights that anticipatory prescribing is not always viable and (re)assessments can be significantly delayed. To facilitate timely reviews, community nurses require easy and rapid access to prescribers in hours and out of hours.^26,46,55,56^ Two-way communication can be facilitated with professional-only bypass telephone numbers, reducing delays. The adoption of electronic prescribing and electronic permission to administer forms can also increase the efficiency, safety, and transfer of information between services.^26^ This is not yet standardised practice in the UK.

Inadequate continuity of care was a recurring contributory factor in many incidents, consistent with previous research.^57–60^ Harmful outcomes were mitigated when clinicians and teams identified and addressed missing or incomplete prescriptions, ‘permission to administer’ charts, and problems with record keeping. Informational continuity of care across services and between in-hours and out-of-hours care providers was a concerning issue contributing to patient harms. Previous studies have stressed the importance of informational and relational continuity in providing consistent, safe, and effective end-of-life care.^3,19,22,45,61–63^ Despite increasing adoption of shared electronic records, informational continuity of care remains fragile when multiple services are involved.^28,45,57,61,64^ The present findings emphasise the importance of cross-organisational structures to provide continuity of care, including clearly defined roles and responsibilities to ensure a suitable symptom control plan is in place and periodically reviewed.

The use of injectable medications relies on skilled professionals being available to administer them at short notice.^12,15,16,21^ This study found the limited availability of staff, often covering large areas, contributed to delays. The skills and knowledge of visiting nurses alongside high workloads, insufficient staffing and continuity of care, contributed to medication administration-related incidents. The present findings support those of previous studies analysing palliative care patient safety incidents.^22–24^ The presence of anticipatory medications in the home does not compensate for overstretched community services,^65,66^ if clinicians are not available to administer medications.^15,52^ Training willing and able family carers to give ‘as required’ injections, with access to phone advice at the time, is an alternative approach being increasingly adopted for a subset of patients in the UK;^27^ this is routine practice in several countries.^9^

Staff factors (such as mistakes) alongside unintuitive and unreliable syringe drivers contributed to injectable medication underdoses and overdoses. Common problems encountered included settings being inadvertently changed or machines turning themselves off between visits. Syringe driver equipment has become more technically complicated to use, partly owing to attempts to correct concerns highlighted in a series of medical device alerts.^67^ As a result, users have to anticipate and mitigate several potential technical issues, often in stressful situations.^24^

### Implications for research and practice

This research shows that injectable medication incidents in the community commonly involve assessment and prescribing issues, alongside medications not being administered in a timely way. Patterns in these primary incidents provide invaluable opportunities to learn from what goes wrong and why. The frequent underestimation of harm in reports, including notable delays in symptom relief going unreported or reported as ‘no harm’ incidents, is problematic as this classification is less likely to get service providers’ attention and stimulate system-wide learning.^68^ Consequently, the authors have conducted a separate study analysing reported ‘no harm’ incidents to explore potential learning.^69^

System-wide targeted interventions are needed to address recurrent contributory factors and mitigate harms. Future syringe driver equipment requires designing in partnership with end users in community settings. Community healthcare systems need to ensure adequate staffing numbers and skills across teams to provide safe and responsive end-of-life symptom control. One related potential intervention, suggested by the study’s public and clinician contributors, is a 2-h system-wide target to visit to administer medication in response to a crisis end-of-life care symptom control care call. Like all targets, this may have desirable and also unintended consequences on wider care delivery and staff wellbeing. However, as providing timely and effective end-of-life symptom relief is a clinical and societal priority this potential lever warrants further research as a pilot intervention.

## Data

The authors are unable to share primary data. These data are held by NHS England and applications for using National Reporting and Learning System data are decided on a case-by-case basis. Permission was granted to Cardiff University through a data-sharing agreement for the duration of this study.
